# Impact of Oxidative Stress in Premature Aging and Iron Overload in Hemodialysis Patients

**DOI:** 10.1155/2016/1578235

**Published:** 2016-10-05

**Authors:** Blanca Murillo-Ortiz, Joel Ramírez Emiliano, Wendy Ivett Hernández Vázquez, Sandra Martínez-Garza, Sergio Solorio-Meza, Froylán Albarrán-Tamayo, Edna Ramos-Rodríguez, Luis Benítez- Bribiesca

**Affiliations:** ^1^Unidad de Investigación en Epidemiología Clínica, Servicio de Hemodiálisis, Unidad Médica de Alta Especialidad (UMAE) No. 1 Bajío, Instituto Mexicano del Seguro Social (IMSS), León, GTO, Mexico; ^2^Departamento de Investigaciones Médicas, Universidad de Guanajuato, León, GTO, Mexico; ^3^Unidad de Investigación Médica en Enfermedades Oncológicas, CMN, SXXI, IMSS, 06720 Ciudad de México, Mexico

## Abstract

*Background.* Increased oxidative stress is a well described feature of patients in hemodialysis. Their need for multiple blood transfusions and supplemental iron causes a significant iron overload that has recently been associated with increased oxidation of polyunsaturated lipids and accelerated aging due to DNA damage caused by telomere shortening.* Methods.* A total of 70 patients were evaluated concomitantly, 35 volunteers with ferritin levels below 500 ng/mL (Group A) and 35 volunteers with ferritin levels higher than 500 ng/mL (Group B). A sample of venous blood was taken to extract DNA from leukocytes and to measure relative telomere length by real-time PCR.* Results.* Patients in Group B had significantly higher plasma TBARS (*p* = 0.008), carbonyls (*p* = 0.0004), and urea (*p* = 0.02) compared with those in Group A. Telomeres were significantly shorter in Group B, 0.66 (SD, 0.051), compared with 0.75 (SD, 0.155) in Group A (*p* = 0.0017). We observed a statistically significant association between relative telomere length and ferritin levels (*r* = −0.37, *p* = 0.001). Relative telomere length was inversely related to time on hemodialysis (*r* = −0.27, *p* = 0.02).* Conclusions.* Our findings demonstrate that iron overload was associated with increased levels of oxidative stress and shorter relative telomere length.

## 1. Introduction

Iron overload is a common complication in patients with renal chronic failure submitted to hemodialysis (HD). It results from the necessity that these patients undergo transfusions of red cells to treat symptomatic anemia, as well as from the administration of enteral and/or parenteral iron supplements [1]. Although it is still controversial, multiple clinical studies have found an association between iron overload and the oxidation of polyunsaturated lipids catalyzed by metallic ions in atherosclerotic disease and in the development of cardiovascular events [2, 3].

There are several studies that demonstrate that iron overload is related with oxidative stress. On both in vivo and in vitro models iron overload allowed us to study the oxidative stress induction through mechanisms which activate the increase on ROS. A study conducted in young women with low iron levels who were given daily supplements of iron found that serum ferritin levels were almost twice the basal levels after 6 weeks of treatment and that body iron was more than twice the basal level. However, plasma levels of malondialdehyde and exhaled ethane increased more than 40% [4].

Chai et al. confirmed that the hematopoietic inhibitory effects of iron overload in an iron-overloaded mouse model were parallel to clinical conditions. Secondly, its related mechanism was investigated. It was demonstrated that iron overload increased the ROS levels of HSPCs through the NOX4/ROS/P38 MAPK signaling pathways [5].

In an experimental study it was observed that taurine supplementation reduces oxidative stress and improves cardiovascular function in an iron-overload murine model [6]. The therapeutic effects of resveratrol in murine iron-overload models that showed cardiac iron overload, increased oxidative stress, altered Ca^2+^ homeostasis, and myocardial fibrosis resulting in heart disease have been recently reported, as well as increased nuclear and acetylated levels of FOXO1 with corresponding inverse changes in SIRT1 levels in the heart which are corrected by resveratrol therapy. Also they demonstrated that the iron mediated pathological effects on human cardiomyocytes and cardiofibroblast were prevented by resveratrol [7].

It also has been shown that iron-overloaded rats had significant increases in malonyl-dialdehyde (MDA), a marker of lipid peroxidation, and nitric oxide (NO) in liver and spleen compared to control group. The effects of iron overload on lipid peroxidation and NO levels were significantly reduced with the administration of curcumin (*p* < 0.05). Furthermore, the endogenous antioxidant activity in liver and spleen was also significantly decreased in chronic iron overload and after administration of curcumin was completely restored [8]. Sripetchwandee et al. demonstrated the first evidence of the effect of combining iron chelator therapy and antioxidants (deferiprone and N-acetylcysteine, resp.) for 4 weeks on the cerebral iron-overload inducted disfunction on Wistar rats which restored completely the cerebral function [9].

The oxidation of polyunsaturated fatty acids generates MDA and 4-hydroxyalkenals; MDA can be measured by TBARS test. Iron-induced oxidative stress may be key determinant in the significant increase of 8-OH-G, 2-hydroxyadenine, and 8-hydroxyadenine adducts [10]. These lesions, caused by hydroxyl radical attack, could significantly increase DNA damage as in the telomere region and/or impair its repair. Telomeres are the specialized DNA structures located at the end of eukaryotic chromosomes and consist of tandemly repeated DNA sequences. Telomeres shorten with each cell division, and it is well known that telomere length in peripheral blood mononuclear cells (PBMCs) decreases with age [11].

The telomere shortening rate is increased by oxidative stress. Boxall et al. suggest that the length of time on dialysis is independently associated with increased telomere shortening in HD patients and hypothesize that this is caused by the cumulative exposure of DNA to oxidative stress [12]. Telomere attrition, expressed in leukocytes (WBCs), can serve as a biomarker of cumulative oxidative stress and inflammation.

Chronic oxidative stress accelerates cellular aging, while telomere shortening has been associated with hypertension, endothelial dysfunction, atherosclerosis, and cardiovascular mortality [13]. A recent study showed that telomeres are shorter in patients with a diagnosis of DM2 with more years of evolution, compared with healthy subjects of the same age. The time of duration of the disease suggests a parallel and progressive increase of inflammation and oxidative stress that plays a direct role in telomere shortening [14].

With aging, the renal function decreases, showing an evidently lower glomerular flow rate. It has been shown that telomere shortening occurs first in cells of the cortex before it occurs in cells of the renal medulla [15]. The increased oxidative stress caused by iron overload may induce this telomere shortening, and this in turn may contribute to renal diseases such as glomerulosclerosis. Measuring telomere length provides evidence of the aging that occurs as a result of the oxidative stress caused by high body iron levels.

Sullivan was the first to suggest (1981) that the high incidence of cardiac disease may be related to elevated serum iron levels [16]. Subsequent investigations provided evidence that high levels of body iron may actually increase the risk of cardiovascular disease. Kiechl and colleagues found a strong correlation between the serum levels of body iron and the probability of developing new atherosclerotic carotid lesions [17].

The Rotterdam study indicated that high levels of body iron, evidenced by a serum ferritin concentration of 200 *μ*g/L or greater, almost double the risk of acute myocardial infarction in elderly patients. In a number of patients in the Bruneck study, serum ferritin and LDL cholesterol showed synergistic effects associated with the progression of carotid atherosclerosis, suggesting that iron promotes lipid peroxidation [18]. Increased oxidative stress and inflammation are associated with atherosclerotic coronary artery disease in hemodialysis patients [19].

Patients with end stage renal disease have a markedly increased risk of presenting cardiovascular complications [20]. Recent evidence shows that there is a strong association between telomere shortening and heart failure [21]. Iron can contribute to cardiovascular complications through its oxidative effects on low-density lipoproteins and its induction of endothelial dysfunction [22–26].

This information has been used recently to enlighten the mechanisms and provide experimental bases to achieve new target therapies on the treatment of the complications generated by the iron overload and on one of its consequences: the oxidative stress. The purpose of our study was to evaluate the effect of iron overload on lipid oxidation and telomere length, as well as the incidence and/or progression of coronary artery disease compared with patients without iron overload, with nephropathy and undergoing renal replacement therapy in the hemodialysis program of our hospital unit.

## 2. Subjects and Methods 

We included a total of 70 patients with nephropathy undergoing renal replacement therapy in the hemodialysis program of the High-Specialty Medical Unit No. 1 Bajío. The patients were of both genders and older than 18 years of age; 35 of them were volunteers with ferritin levels higher than 500 ng/mL (Group A) and 35 were volunteers with ferritin levels below 500 ng/mL (Group B), who were evaluated concomitantly. Patients with immune disorders and the habit of smoking and alcoholism were not included. A sample of venous blood was taken in order to extract DNA from leukocytes and to measure telomere length by real-time PCR. Serum levels of ferritin and oxidation markers were also determined. All patients underwent transthoracic echocardiography. This protocol was approved by the local bioethics committee and a written informed consent was obtained from each volunteer.

### 2.1. Measurement of Biochemical Parameters

Serum levels of glucose were determined using the glucose oxidase-peroxidase method (Biosystems, Spain). Uric acid, urea, creatinine, cholesterol, and triglycerides were estimated using enzymatic methods (STANBIO Laboratory, Boerne, TX, USA). Ferritin levels in plasma were determined with an automated analyzer using dry chemistry technique. The analyzer is Johnson & Johnson's E60 IQ which is reported in units of the IS (ng/mL).

### 2.2. Telomere Measurement

DNA samples were extracted from white blood cells. The ratio of telomere repeat copy number to a single gene copy number (T/S) was determined by a previously described modified version of the quantitative real-time PCR telomere assay [27]. We performed PCR amplification with oligonucleotide primers designed to hybridize to the TTAGGG and CCCTAA repeats. The final concentrations of reagents in the PCR were 0.2 SYBR Green I (Molecular Probes), 15 mM Tris-HCl pH 8.0, 50 mM KCl, 2 mM MgCl_2_, 0.2 mMeach dNTP, 5 mM DTT, 1% DMSO, and 1.25 U AmpliTaq Gold DNA polymerase. The final telomere primer concentrations were tel 1,270 nM and tel 2,900 nM. The final* 36B4 *(single copy gene) primer concentrations were 36B4u, 300 nM, 36B4d, and 500 nM. The primer sequences (written 5′ → 3′) were tel 1, GGTTTTTGAGGGTGAGGGTGAGGGTGAGGGTGAGGGT, tel2, TCCCGACTATCCCTATCCCTATCCCTATCCCTATCCCTA, 36B4u, CAGCAAGTGGGAAGGTGTAATCC, 36B4d, and CCCATTCTATCATCAACGGGTACAA. All PCRs were performed on a LightCycler® 1.5 (Roche). The thermal cycling profile for both amplicons began with a 95°C incubation for 3 min to activate the AmpliTaq Gold DNA polymerase. The telomere PCR conditions were 40 cycles of 95°C for 15 s and 54°C for 2 min; for* 36B4* PCR, they were 40 cycles of 95°C for 15 s and 58°C for 1 min. The LightCycler 1.5 (Roche) was then used to generate the standard curve for each run and to determine the dilution factors of standards corresponding to the amounts of T and S in each sample.

### 2.3. Measurement of Lipid Peroxidation and Oxidized Protein

Malondialdehyde levels and carbonyls content were quantified as we previously described [28]. The MDA levels were determined with the thiobarbituric acid-reactive substances (TBARS) assay using 30 *μ*L of sera, whereas the carbonyl content was measured using 5 *μ*L of sera.

### 2.4. Cardiovascular Evaluation

#### 2.4.1. Echocardiogram

The echocardiogram was performed with a Hewlett Packard Sonos 5500 equipped with an electronic 3.5 MHz phased array probe and an 8 MHz linear array probe. The diameters of the left atrium, left ventricle, right ventricle, interventricular septum, and posterior wall of the left ventricle were measured. The image analysis was performed according to the criteria of the American Society of Echocardiography. The left ventricular mass was calculated using the Devereux method. The results of the echocardiographic studies were recorded on a CD for subsequent assessment by two experienced echocardiographers who determined by consensus whether the patient had ischemia or myocardial infarction.

### 2.5. Statistics

To determine the differences between groups, we used Student's *t*-test for independent samples. The Pearson correlation coefficient (*r*) was used to assess the association between relative telomere length and other variables. All data are presented as mean ± SE, with *p* < 0.05 as a cut-off for statistical significance.

## 3. Results

### 3.1. General Characteristics

We included 70 patients of both genders with nephropathy on renal replacement therapy, 35 of which had ferritin levels below 500 ng/mL, aged 46.48 ± 16.9 years (Group A), and the other 35 had ferritin levels higher than 500 ng/mL, aged 45.34 ± 16.57 years (Group B). Patients were receiving i.v. iron dextran at an average of ≥400 mg/month. Patient characteristics of the different groups are shown in Table 1.

### 3.2. Free Iron and Oxidative Stress: Lipid Peroxidation and Oxidized Protein

Serum levels of ferritin and oxidation markers were determined. The levels of ferritin were 308 ± 145 ng/dL (Group A) versus 3224 ± 2078 ng/dL (Group B), *p* < 0.001. Patients with ferritin levels higher than 500 ng/mL had significant differences with higher levels of TBARS (11.7 ± 4.6 versus 9.4 ± 2.2 nmoles/mL, *p* = 0.008), carbonyls (27.2 ± 5.2 versus 22.5 ± 5.4 ng/*μ*L, *p* = 0.0004), and urea (131.10 ± 50.77 versus 104.23 ± 46.36 mg/dL, *p* = 0.02) compared with patients with ferritin levels below 500 ng/mL (Group A) (Table 2).

**Figure 1:**
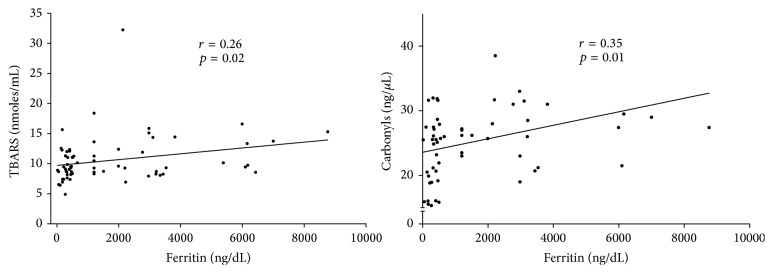
Serum TBARS and carbonyls were positively associated with ferritin levels in patients with nephropathy undergoing renal replacement therapy in hemodialysis (*n* = 70).

**Figure 2:**
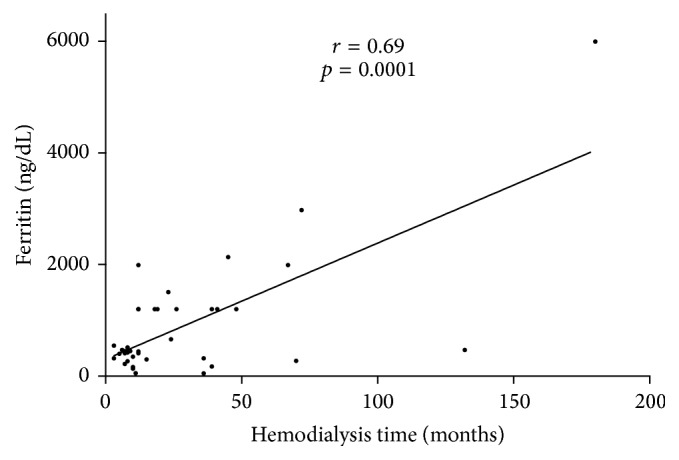
The relationship between ferritin (ng/dL) and length of time on hemodialysis in all subjects (*n* = 70).

Interestingly serum TBARS and carbonyls were positively associated with ferritin levels in all subjects (*r* = 0.26, *p* = 0.02, *r* = 0.35, *p* = 0.01, resp.); the relationship is shown in Figure 1. A length of time under hemodialysis demonstrated significant relationship with higher ferritin levels (*r* = −0.27, *p* = 0.02) as well (Figure 2).

### 3.3. Relative Telomere Length

**Figure 3:**
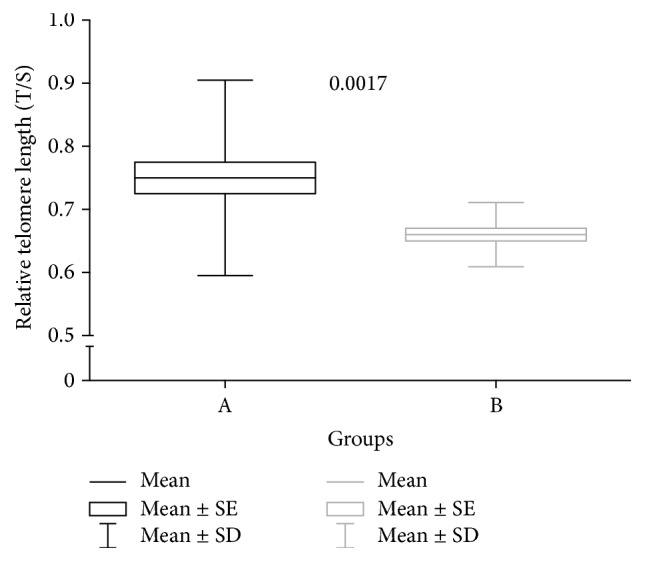
Comparison of relative telomere lengths (T/S) between groups.

**Figure 4:**
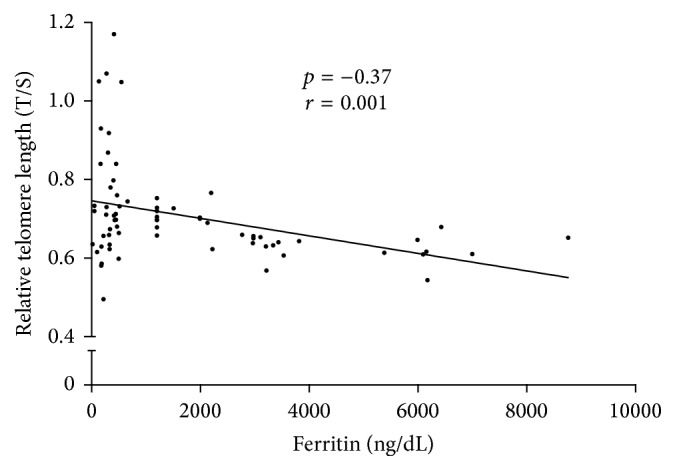
The relationship between relative telomere length (T/S) and ferritin.

**Figure 5:**
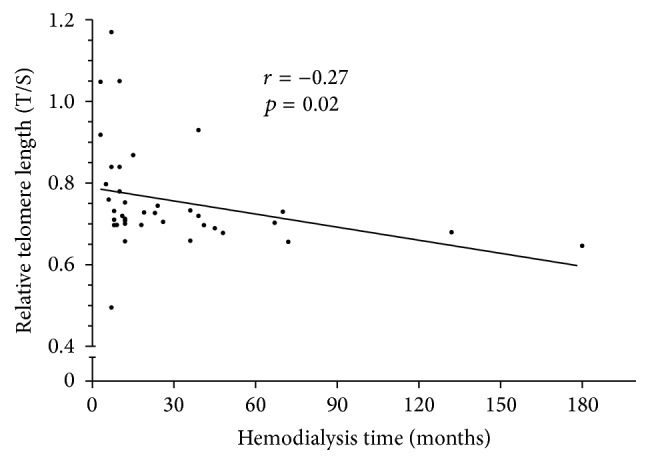
The relationship between relative telomere length (T/S) and time under hemodialysis.

A sample of venous blood was taken in order to extract DNA from leukocytes and to measure relative telomere length by PCR in real time. The length of telomeres was markedly shorter in the group with higher ferritin levels. Telomeres were significantly shorter in Group B than in Group A. The relative telomere length (T/S) in patients with higher ferritin levels was 0.66 (SD, 0.051), versus 0.75 (SD, 0.155) in controls (*p* = 0.0017) (Figure 3). We observed a statistically significant association between relative telomere length and ferritin levels (*r* = −0.37, *p* = 0.001) (Figure 4). We did not observe an inverse significant correlation between relative telomere length and age (*r* = 0.01, *p* = 0.51). Relative telomere length (T/S) was inversely related to the time under hemodialysis (*r* = −0.27, *p* = 0.02) (Figure 5).

### 3.4. Iron and Cardiovascular Disease

We found through the analysis of echocardiographic parameters that hypertrophy of the left ventricle, related to the Left Ventricular Mass Index (LVMI), was higher in the high ferritin level group (29 patients, 82.86%, versus 8 patients, 22.86%, *p* = 0.01). The results of echocardiographic examination are summarized in Table 3.

In this study, the shortening of telomeres was associated with a significant increase in left ventricular mass (*r* = 0.40, *p* = 0.01). The linear regression analysis between serum levels of ferritin with left ventricular mass observed was *p* = 0.002.

## 4. Discussion

The results of this study suggest that higher ferritin levels are associated with increased telomere shortening in hemodialysis patients. On this matter oxidative stress and inflammation are well-established key factors in the pathogenesis of atherosclerosis and vascular disease among chronic kidney disease patients [29].

Moreover, oxidative stress has also been related to immune system dysregulation in patients with uremia, indicated by increased oxidative biomarkers and activation of circulating leukocytes [30]. Uremia has been determined as an important factor in premature aging in patients with end stage renal disease [31]. Importantly, in our study serum urea levels were significantly higher in the group with higher ferritin levels (Group B); this metabolic condition induces various abnormalities in the patient, increasing the risk of developing uremic encephalopathy and other complications associated with poor prognosis.

Iron overload is a common complication in patients submitted to hemodialysis; the process of lipid peroxidation is catalyzed by iron and results in the formation of peroxyl radicals. We found a strong correlation between iron overload and increased oxidative biomarkers. The group with higher ferritin levels showed more telomere shortening; relative telomere length (T/S) in patients with higher ferritin levels was 0.66 (SD, 0.051), versus 0.75 (SD, 0.155) in controls (*p* = 0.0017); the telomere shortening rate was increased by oxidative stress. There is no immediate explanation of this phenomenon, but the relative increase in oxidative markers observed in patients with iron overload could be a contributing factor. The most significant finding of our study was the observation that hemodialysis patients with higher ferritin levels (Group B) showed increased telomere shortening.

Oxidative stress, in association with chronic inflammation, has been suggested as a possible contributory factor for this increase in mortality as well [32]. The increased oxidative stress caused by iron overload may induce this telomere shortening, which may contribute to renal diseases such as glomerulosclerosis, and prevent renal regeneration [33, 34].

Patients with chronic kidney disease have significantly increased morbidity and mortality from cardiovascular disease. Previous studies demonstrated a potential role of anemia in the pathogenesis of left ventricular hypertrophy [35]. Moreover several previous studies have documented that key cardiovascular diseases that increase with age (coronary atherosclerosis, arterial stiffening, increased carotid intima/media thickness, and clinically overt cardiovascular disease events such as myocardial infarction and stroke) are all associated with shortened telomere length. Likewise, vascular risk factors such as smoking, body mass index, and hypertension are inversely associated with telomere length [36].

Iron-overload cardiomyopathy is a prevalent cause of heart failure on a worldwide basis and is a major cause of mortality and morbidity in patients with secondary iron overload. In this study the analysis of echocardiographic parameters showed that higher ferritin levels in hemodialysis patients are associated with a significant increase in left ventricular mass which has been associated with higher cardiovascular risk in other studies. Murine iron-overload models showed cardiac iron overload, increased oxidative stress, altered Ca^2^ homeostasis, and myocardial fibrosis resulting in heart disease [37].

Premature cardiovascular disease is a significant cause of morbidity and mortality [38], while end stage renal disease, and particularly its treatment with hemodialysis, is a condition of increased oxidative stress [39, 40].

Iron-induced oxidative stress plays a fundamental role in the pathogenesis of iron-overload mediated heart disease. The basic molecular mechanism of iron-overload cardiomyopathy has not been elucidated and strategies to treat this global epidemic are limited. Iron overload in humans leads to an advanced cardiomyopathy [41–44] and the development and validation of preclinical models of iron-overload cardiomyopathy are important for the discovery of new therapies [45, 46].

Monitoring the serum levels of ferritin has several benefits for patients with renal disease that are at increased risk of cardiovascular events, such as limiting the damage caused by oxidative stress [47, 48] and thus allowing the initiation of appropriate preventive therapeutic measures such [49] as the use of iron chelators. Telomere attrition, expressed in white blood cells (WBCs), can serve as a biomarker of the cumulative oxidative stress.

## 5. Conclusions

Our findings demonstrate that iron overload was associated with increased levels of oxidative stress and shorter relative telomere length. Identifying factors that accelerate the aging process in end stage renal disease can provide important therapeutic targets to revert this process. High levels of ferritin are related to signs of increased oxidative stress as reflected by increased TBARS and carbonyl levels in hemodialysis patients. Iron overload was found to be a major contributing factor to left ventricular hypertrophy. In addition iron overload is a potential therapeutic target to prevent premature aging and iron chelation agents may limit the increased oxidative stress in these patients.

## Figures and Tables

**Table 1:** Demographic and clinical characteristics in patients with high ferritin levels (Group A) and low ferritin levels (Group B).

	Group A (*n* = 35)	Group B (*n* = 35)	*p*
	*n* (%)	*n* (%)	
Female (%)	51.42 (18)	51.42 (18)	Ns
Male (%)	48.57 (17)	48.57 (17)	Ns
Age in years	45.4 ± 16.6	46.5 ± 16.9	0.77
Duration on HD (months)	40.87 ± 41.65	20.17 ± 29.00	0.01^*∗*^
Diabetes (%)	68.57 (24)	51.42 (18)	0.22
Hypertension (%)	11.43 (4)	17.14 (6)	0.73
Other (%)	20 (7)	31.42 (11)	0.41

Data shown as mean ± SD. Student's *t*-test was used to determine the differences.

**Table 2:** Comparison of biochemical indices of iron status and oxidative markers between groups.

	Group A (*n* = 35)	Group B (*n* = 35)	*p*
	Mean ± SD	Mean ± SD	
Uric acid (mg/dL)	5.83 ± 1.04	5.45 ± 1.44	0.24
Creatinine (mg/dL)	7.81 ± 2.42	8.97 ± 2.65	0.06
Urea (mg/dL)	104.2 ± 46.3	131.10 ± 50.7	0.02^*∗*^
Glucose (mg/dL)	100.9 ± 34.1	90.7 ± 30.3	0.18
Hemoglobin (g/dL)	11.23 ± 1.93	12.54 ± 1.85	0.01^*∗*^
Ferritin (ng/mL)	308 ± 145	3224 ± 2078	<0.0001^*∗*^
Iron (*μ*g/dL)	78.5 ± 38.7	152.7 ± 86.1	<0.0001^*∗*^
Iron saturation percentage (%)	48.3 ± 33.5	82.8 ± 39.2	0.0002^*∗*^
TBARS (nmol/mL)	9.4 ± 2.2	11.7 ± 4.6	0.008^*∗*^
Carbonyls (ng/*μ*L)	22.5 ± 5.4	27.2 ± 5.2	0.0004^*∗*^

^*∗*^Data shown as mean ± SD. Student's *t*-test was used to determine the differences.

**Table 3:** Echocardiographic examination results.

Parameter	(*n* = 70)
LV mass (gr)	186.6 ± 67.4
LV systolic pressure (mm)	32.2 ± 5.6
LV diastolic pressure (mm)	50.7 ± 7.0
Ejection fraction (%)	65.2 ± 6.1
Fractional shortening (%)	36.6 ± 5.9
